# Cytokine Profiles in Asthma Families Depend on Age and Phenotype

**DOI:** 10.1371/journal.pone.0014299

**Published:** 2010-12-13

**Authors:** Katrin Pukelsheim, Tobias Stoeger, David Kutschke, Koustav Ganguly, Matthias Wjst

**Affiliations:** 1 Institute of Lung Biology and Disease, Helmholtz Zentrum München, Munich, Germany; 2 Department of Environmental and Occupational Health, Graduate School of Public Health, University of Pittsburgh, Pittsburgh, Pennsylvania, United States of America; The Johns Hopkins University, United States of America

## Abstract

**Background:**

Circulating cytokine patterns may be relevant for the diagnosis of asthma, for the discrimination of certain phenotypes, and prognostic factors for exacerbation of disease.

**Methodology/Principal Findings:**

In this study we investigated serum samples from 944 individuals of 218 asthma-affected families by a multiplex, microsphere based system detecting at high sensitivity eleven asthma associated mediators: eotaxin (CCL11), granulocyte macrophage stimulating factor (GM-CSF), interferon gamma (IFNγ), interleukin-4 (IL-4), IL-5, IL-8, IL-10, IL-12 (p40), IL-13, IL-17 and tumor necrosis factor alpha (TNFα). Single cytokine levels were largely similar between asthmatic and healthy individuals when analysing asthma as single disease entity. Regulatory differences between parental and pediatric asthma were reflected by six of the eleven mediators analyzed (eotaxin, IL-4, IL-5, IL-10, IL-12, TNFα). IL-12 (p40) and IL-5 were the best predictor for extrinsic asthma in children with an increased odds ratio of 2.85 and 1.96 per log pg/ml increase (IL-12 (p40): 1.2–6.8, p = 0.019, and IL-5: 1.2–2.5, p = 0.025). Frequent asthma attacks in children are associated with elevated IL-5 serum levels (p = 0.013). Cytokine patterns seem to be individually balanced in both, healthy and diseased adults and children, with various cytokines correlating among each other (IL-17 and IFNγ (r_s_ = 0.67), IL-4 and IL-5 (r_s_ = 0.55), IFNγ and GM-CSF (r_s_ = 0.54)).

**Conclusion/Significance:**

Our data support mainly an age- but also an asthma phenotype-dependent systemic immune regulation.

## Introduction

Asthma is the most common serious chronic lung disease and shows increasing incidence in industrialized as well as in developing countries, afflicting around 150 million people worldwide [1]. With airway hyper-responsiveness being the physiological hallmark of asthma, it is also characterized by chronic inflammation of the respiratory tract, allergen-specific IgE production, infiltration of eosinophils, the recruitment of T cells into the airways, and alterations in the fine balance between type 1 helper T lymphocytes (T_h_1) and type 2 helper T lymphocytes (T_h_2) responses towards T_h_2 bias [2].

T_h_2 cells secret a panel of cytokines with several overlapping functions including Interleukin-4 (IL-4), IL-5, IL-13, and granulocyte-macrophage colony stimulating factor (GM-CSF). By mediating differentiation of the T_h_2 subpopulation and eosinophils, as well as modulating B-cell proliferation and IgE switching, the T_h_2 cytokines are thought to play a prominent role in asthma [3], [4]. The sentinel T_h_1 cytokine, interferon gamma (IFNγ) and IL-12 reciprocally stimulate their production and function during cell-mediated immunity and development of naïve T lymphocytes into T_h_1 cells. Evidence suggests a contributory role of T_h_1 cells and their cytokines in asthmatic inflammation and airway hyperresponsiveness [5], [6]. The T cell subset of regulatory T cells (Treg) acts by expressing immunosuppressive cytokines, such as IL-10, of which impaired production has been reported in asthmatic patients [7]. Moreover, the lymphocyte lineage T_h_17 is increased in inflamed airways [8] and characterised by the production of IL-17. This pro-inflammatory cytokine is capable of causing the release of other pro-inflammatory cytokines, such as IL-8, tumor necrosis factor alpha (TNFα), and GM-CSF, which have been associated with asthma in murine models [9], in humans [10], [11], or with disease severity [12], [13], [14], [15].

Asthma – probably the most heterogeneous lung disease – may also demonstrate systemic patterns outside the respiratory system. Determination of serum cytokine levels in asthmatic patients could have potential utility in diagnosis of asthma and certain phenotypes, in prediction of attacks, and treatment choice. Although there are numerous in vitro, as well as in vivo data from animal studies, to date no information of large scale clinical studies exist that examines a correlation between circulating cytokine levels and asthma phenotypes or exacerbations of disease in different age groups.

Thus, the present study aimed at investigating circulating cytokine and chemokine levels of healthy and asthmatic children as well as their parents. We hypothesized that one of the analyzed cytokines and chemokines, or a combination is suited as a serological marker for asthma or certain phenotypes. To test our hypothesis we measured serum concentrations of eleven cytokines and chemokines that were recently described to play an important role in asthma pathogenesis: IL-4, IL-5, IL-8, IL-10, IL-12 (p40), IL-13, IL-17, TNFα, GM-CSF, eotaxin, and IFNγ.

## Materials and Methods

### Study Population

218 core families were selected during two study stages. The sample is identical to previous reports [16], [17], [18] describing explicitly the selection process, study participants and their clinical characteristics. In the first stage, 97 families consisting of at least two children with confirmed clinical asthma were selected for an initial genome-wide linkage scan [18]. The families were collected mainly in pediatric university clinical centers in Germany and Sweden. At least two children with confirmed clinical asthma were required, and prematurity or low birth weight of the children was excluded, along with any severe pulmonary disease other than asthma. In the second study stage, another set of asthma sib pair families was selected. Three university hospitals and six pediatric pulmonary practices carried out an identical phenotyping procedure as during the first stage.

The 218 families comprised 944 individuals, of whom 424 were parents with a mean age of 40 years and 499 were children with a mean age of 11 years; 21 individuals had insufficient clinical documentation and were excluded from further analysis. 275 of the children were male, 224 were female and 443 had physician diagnosed asthma. The youngest child diagnosed with asthma was 4 years old and the oldest child was 34 years old. Fifty-eight percent of the children with asthma were boys. Asthma was initially defined by clinical history and validated later by interview questions. In addition to a clinical asthma diagnosis, all affected children over age 3 had a history of at least 3 years of recurrent wheezing and with no other airway diseases diagnosed.

The phenotyping procedure contained detailed interviews of every family member, skin prick tests (SPT) of frequent allergens, blood samples (for IgE and allergen-specific IgE (RAST) measurements, eosinophil count), peak flow tests for a period of 10 days and dust collection at patients home. Total IgE was determined with an ELISA (Pharmacia Diagnostics, Uppsala, Sweden). SPT and RAST assays included several pollens, animal furs, mould, and house dust mite allergens (ALK-SCHERAX, Hamburg, Germany). Atopic (extrinsic) asthma was defined if patients demonstrated at least one positive skin prick test reaction or one increased specific IgE. Pulmonary function tests were performed by forced flow volume tests and bronchial challenge was done by a defined increasing methacholine exposure. Written consent was given by all study participants. The parent/guardians of all participants under 18 gave informed consent too. All study methods were approved in 1995 by the Ethics Commission of “Nordrhein-Westfalen” and again in 2001 by “Bayerische Landesärztekammer München”.

### Cytokine Assays

Concentration of eotaxin, GM-CSF, IFN-γ, IL-4, IL-5, IL-8, IL-10, IL-12 (p40), IL-13, IL-17, TNF-α in the serum samples was quantified using a custom-made MILLIPLEX™ MAP (multiple analyte profile) Human Cytokine/Chemokine Panel (Millipore, Schwalbach/Ts, Germany). The assay was performed according to the recommendations of the manufacturer using a 8-point standard curve for every cytokine. Samples were analyzed on a Luminex 100 device (BioRad, München, Germany) and the data were evaluated using the Bio-Plex Manager software (BioRad). Standards and internal controls were reported as means of duplicate measurements. Samples were analyzed in single measurements, since the MILLIPLEX™ MAP assay shows good precision in pre-tests, with a stated intra-assay mean coefficient of variance (CV) of 4.5% to 10.4%. The lower detection limits for every cytokine and numbers of samples that were below the lower detection limit and above the upper detection limit are listed in Table S1 (online data supplement). Values below the detection limit were set to the half of the detection limit and to 3000 pg/ml if exceeding the maximum concentration of 3000 pg/ml.

### Analysis

Statistical analyses were performed using R software. Following a descriptive analysis using cross-tabulation and distribution of single variables, linear and logistic regression analysis was used for determination of effect estimates.

## Results

The serum concentrations of the 11 cytokines and chemokines IL-4, IL-5, IL-8, IL-10, IL-12 (p40), IL-13, IL-17, TNFα, GM-CSF, Eotaxin, and IFNγ could be obtained from 944 individuals (Figure S1) from 218 families, of whom 424 were parents and 499 were children. Main demographic and clinical characteristics of study participants were summarized in previous reports [16], [17], [18]. In table 1 healthy and asthmatic parents are compared as well as healthy and asthmatic children. Except of IL-8 (p = 0.012) and IL-10 (p = 0.045) in the adult group, none of the cytokine concentrations in serum differ significantly between healthy and diseased individuals. However, mean cytokine levels for six of the eleven cytokines are significantly different between asthmatic children and asthmatic adults. Values for Eotaxin (p = 0.006), IL-10 (p = 0.002), and TNFα (p = 0.038) are higher in parents than in children. In contrast IL-4 (p<0.001), IL-5 (p<0.001), and IL-12 (p40) (p = 0.001) serum levels are lower in diseased adults compared to diseased children.

**Table 1:** Case control analysis of parents and children with and without asthma.

Cytokines	descriptors	parents healthy n = 321	parents asthma n = 103	children healthy n = 56	children asthma n = 443	pediatric versus parental asthma
eotaxin	median [pg/ml]	117.2	105.3	92.1	88.2	
	iqr	80–164	73–150	72–116	65–121	
	**P**		***0.141*** *		***0.516*** **	***0.006*** ***
	*Bonf. P*		*1*		*1*	*0.072*
GM-CSF	median	73.5	82.8	121.6	77.0	
	iqr	25–228	25–290	29–334	29–202	
	**P**		***0.699***		***0.167***	***0.620***
	*Bonf. P*		*1*		*1*	*1*
IFNγ	median	9.3	9.9	17.5	11.5	
	iqr	3–28	3–30	4–41	5–30	
	**P**		***0.593***		***0.212***	***0.607***
	*Bonf. P*		*1*		*1*	*1*
IL-4	median	0.3	0.3	16.2	12.8	
	iqr	0–10	0–16	1–240	0–65	
	**P**		***0.976***		***0.118***	***0.000***
	*Bonf. P*		*1*		*1*	*0.000*
IL-5	median	0.0	0.0	0.5	0.6	
	iqr	0–0	0–1	0–3	0–2	
	**P**		***0.369***		***0.941***	***0.000***
	*Bonf. P*		*1*		*1*	*0.000*
IL-8	median	28.4	51.9	39.3	33.5	
	iqr	10–84	18–197	17–82	13–117	
	**P**		***0.012***		***0.531***	***0.075***
	*Bonf. P*		*0.144*		*1*	*0.900*
IL-10	median	1.6	2.4	4.5	3.5	
	iqr	1–4	1–6	2–14	2–8	
	**P**		***0.045***		***0.168***	***0.002***
	*Bonf. P*		*0.54*		*1*	*0.024*
IL-12(p40)	median	22.5	9.0	59.7	42.0	
	iqr	5–64	5–63	7–123	5–108	
	**P**		***0.833***		***0.447***	***0.001***
	*Bonf. P*		*1*		*1*	*0.012*
IL-13	median	0.2	0.2	3.4	1.0	
	iqr	0–5	0–8	0–8	0–6	
	**P**		***0.816***		***0.063***	***0.275***
	*Bonf. P*		*1*		*0.756*	*1*
IL-17	median	9.1	10.7	12.1	7.8	
	iqr	2–28	2–30	3–33	2–26	
	**P**		***0.934***		***0.165***	***0.737***
	*Bonf. P*		*1*		*1*	*1*
TNFα	median	4.9	5.7	6.4	6.2	
	iqr	4–8	4–8	4–10	5–10	
	**P**		***0.374***		***0.694***	***0.038***
	*Bonf. P*		*1*		*1*	*0.456*

*parental asthma vs no asthma,

**pediatric asthma vs no asthma,

***pediatric vs parental asthma.

Descriptors of cytokines and chemokines include the median serum concentration in pg/ml, the range of first and third quartile (iqr), the p-value in bold type (P), and Bonferroni adjusted p-values in italics (Bonf. P) evaluated through non-parametric Wilcoxon test after log-transformation. Numbers of samples (n) of the respective group are given in the upper row.

**Figure 1:**
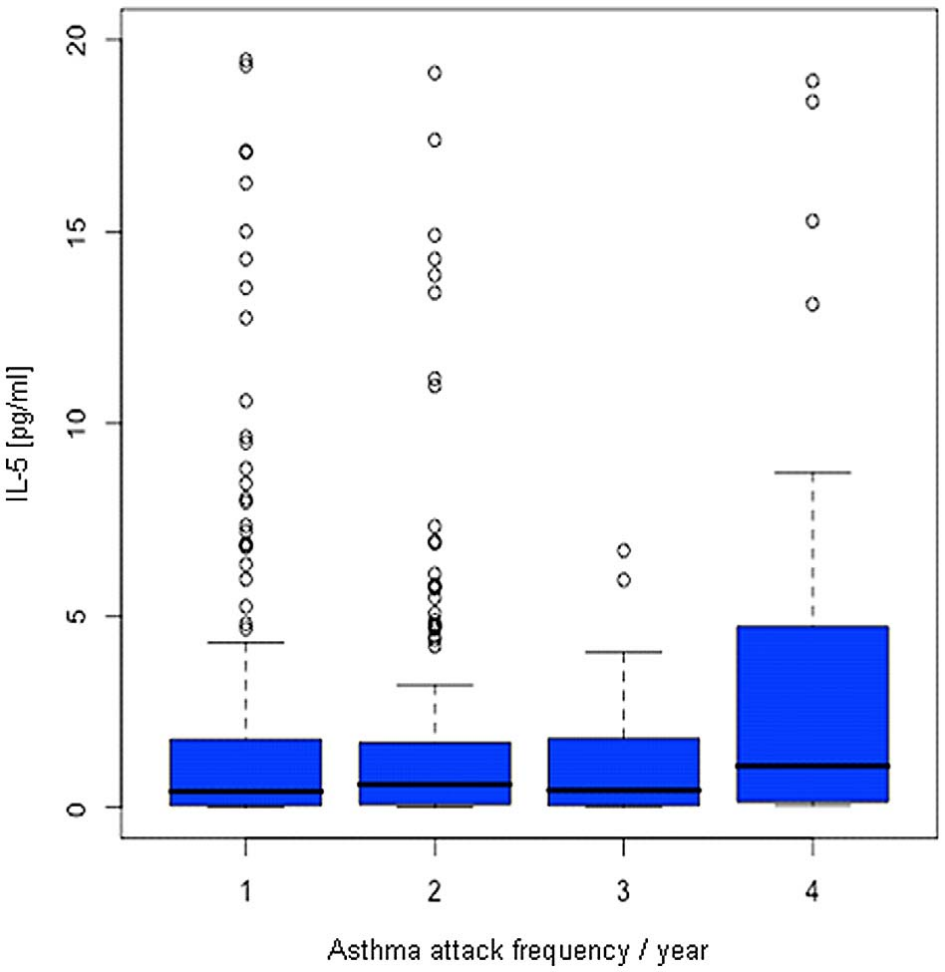
Box plot of serum IL-5 concentrations and asthma attack rates in one year. Only asthmatic children (n = 443) were included in this analysis. The numbers of asthma attacks occurring in the last year were classified into continuous categories from one to four. Numbers of samples of the respective groups are 213 (group 1), 175 (group 2), 26 (group 3), and 29 (group 4). Children with the most frequent asthma attacks had the highest serum levels of IL-5 (p = 0.013).

Results of serum cytokine measurements of the asthmatic children and distinct clinical parameters were then further analyzed in a regression model. In table 2 regression coefficients and p-values are calculated using logarithmic values. Of the blood cell counts, lymphocyte and eosinophil counts are inversely associated to their cooperating chemokines (lymphocyte numbers and IL-8: r = −0.019, p = 0.011; eosinophil numbers and eotaxin: r = −0.432, p = 0.033). Furthermore IL-5 is positively associated with eosinophilia (r = 0.119, p = 0.050), while IFNγ/IL-5 demonstrates an inverse association to eosinophil counts (r = −0.119, p = 0.030). Regression analysis of serum cytokine levels to established clinical parameters of asthma, such as current lung function (FEV1) and bronchial hyperreactivity (BHR), showed weak results. Of all cytokines measured only the serum concentrations of IL-4 (r = 0.016, p = 0.013), is associated with the current lung function FEV1. However, in spite of a remarkable result for IL-12 (p40) (p = 0.056), none of the eleven circulating cytokines is related to BHR. But, classifying the number of asthma attacks occurring in the last year to categories from one to four, revealed that frequent asthma attacks in children are associated with higher IL-5 serum levels (p = 0.013, Figure 1).

**Table 2:** Regression analysis of serum cytokines and clinical characteristics.

logarithmic values	lymphocyte counts	eosinophil counts	total IgE	IgE for D1	IgE for GX1	attack frequency	asthma onset	FEV1	BHR
eotaxin	0.002	−0.432	−0.327	−0.595	−0.108	−0.123	1.086	−0.019	0.485
	**0.924**	**0.033**	**0.007**	**0.003**	**0.543**	**0.503**	**0.071**	**0.466**	**0.223**
	*1*	*0.396*	*0.084*	*0.036*	*1*	*1*	*0.852*	*1*	*1*
GM-CSF	0.002	0.039	0.037	0.031	0.027	−0.034	0.211	−0.011	−0.106
	**0.813**	**0.549**	**0.322**	**0.623**	**0.632**	**0.546**	**0.260**	**0.221**	**0.411**
	*1*	*1*	*1*	*1*	*1*	*1*	*1*	*1*	*1*
IFNγ	0.002	−0.037	−0.024	0.014	0.013	0.099	0.129	−0.017	0.038
	**0.845**	**0.659**	**0.631**	**0.866**	**0.863**	**0.184**	**0.605**	**0.128**	**0.823**
	*1*	*1*	*1*	*1*	*1*	*1*	*1*	*1*	*1*
IL-4	−0.005	−0.006	0.049	0.038	0.017	0.035	0.131	0.016	−0.014
	**0.282**	**0.907**	**0.076**	**0.404**	**0.671**	**0.395**	**0.354**	**0.013**	**0.892**
	*1*	*1*	*0.912*	*1*	*1*	*1*	*1*	*0.156*	*1*
IL-5	−0.008	0.119	0.143	0.174	0.094	0.118	−0.061	0.012	0.011
	**0.197**	**0.050**	**0.000**	**0.004**	**0.079**	**0.027**	**0.739**	**0.183**	**0.935**
	*1*	*0.600*	*0.000*	*0.048*	*0.948*	*0.324*	*1*	*1*	*1*
IL-8	−0.019	0.093	0.042	−0.064	0.150	−0.149	0.384	0.016	0.231
	**0.011**	**0.212**	**0.338**	**0.377**	**0.019**	**0.022**	**0.076**	**0.335**	**0.365**
	*0.132*	*1*	*1*	*1*	*0.228*	*0.264*	*0.912*	*1*	*1*
IL-10	0.001	−0.137	0.052	−0.030	0.047	0.015	−0.036	0.020	0.246
	**0.951**	**0.159**	**0.353**	**0.745**	**0.566**	**0.860**	**0.903**	**0.111**	**0.191**
	*1*	*1*	*1*	*1*	*1*	*1*	*1*	*1*	*1*
IL-12 (p40)	−0.003	0.072	0.101	0.161	0.032	−0.048	−0.276	0.002	0.306
	**0.762**	**0.374**	**0.036**	**0.043**	**0.646**	**0.509**	**0.249**	**0.829**	**0.056**
	*1*	*1*	*0.432*	*0.516*	*1*	*1*	*1*	*1*	*0.672*
IL-13	0.008	−0.038	0.001	−0.040	−0.008	0.002	−0.014	0.001	−0.002
	**0.150**	**0.474**	**0.981**	**0.438**	**0.857**	**0.964**	**0.930**	**0.908**	**0.989**
	*1*	*1*	*1*	*1*	*1*	*1*	*1*	*1*	*1*
IL-17	−0.010	−0.027	−0.031	−0.006	0.031	0.078	0.068	−0.010	0.187
	**0.189**	**0.706**	**0.466**	**0.937**	**0.623**	**0.219**	**0.750**	**0.283**	**0.189**
	*1*	*1*	*1*	*1*	*1*	*1*	*1*	*1*	*1*
TNFα	−0.020	0.116	−0.058	−0.215	0.023	−0.218	0.875	−0.036	0.684
	**0.253**	**0.500**	**0.569**	**0.243**	**0.888**	**0.154**	**0.086**	**0.180**	**0.091**
	*1*	*1*	*1*	*1*	*1*	*1*	*1*	*1*	*1*
IFNγ/IL-5	0.006	−0.119	−0.133	−0.129	−0.079	−0.035	−0.010	−0.018	0.011
	**0.311**	**0.030**	**0.000**	**0.016**	**0.097**	**0.475**	**0.953**	**0.014**	**0.922**
	*1*	*0.360*	*0.000*	*0.192*	*1*	*1*	*1*	*0.168*	*1*

Depicted are regression coefficients followed by p-values (in bold type) and Bonferroni adjusted p-values (in italics) calculated of the logarithmic values of the cytokines and clinical parameters of asthmatic children (n = 443).

**Figure 2:**
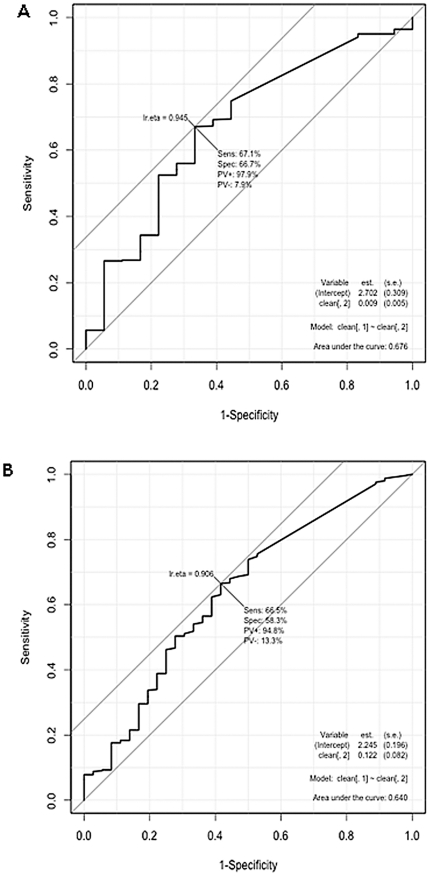
Receiver operating characteristic for the diagnostic value of IL-12 (p40) (A) and IL-5 (B). Only asthmatic children (n = 443) were included in this analysis. Area under the curve of the ROC plot produced a sensitivity of 67.1% and specificity of 66.7% for IL-12(p40), and a sensitivity of 66.5% and a specificity of 58.3% for IL-5 to distinguish between the extrinsic and intrinsic asthma phenotype.

In table 3 the odds ratios (OR) are used to describe the predictive value of serum cytokine levels to asthma subtypes, allergic rhinitis, eczema, actual medication and hospitalization frequency, while only asthmatic children were included in this analysis. Atopic (extrinsic) asthma was defined if patients demonstrated at least one positive skin prick test reaction or one increased specific IgE. IL-12 (p40) is the best predictor for extrinsic asthma in children with an increased OR of 2.85 per log pg/ml increase (1.19–6.84, p = 0.019) or vice versa with a decreased OR of 0.35 (0.15–0.84, p = 0.019) for intrinsic asthma. Moreover atopic and non-atopic asthma phenotypes were significantly influenced by IL-5 levels (OR for atopic asthma 1.96 (1.09–3.52, p = 0.025). A receiver operating characteristic (ROC) revealed a sensitivity of 67.1% for IL-12 (p40) and 66.5% for IL-5 and specificity of 66.7% for IL-12 (p40) and 58.3% for IL-5 serum levels to distinguish between the extrinsic and intrinsic asthma phenotype in children (Figure 2 A and B). The association of serum cytokine levels to other atopic diseases, such as allergic rhinitis and eczema, showed mixed results. For the latter no circulating cytokine was predictive and for allergic rhinitis a strong impact of IL-5 serum levels (OR: 1.42 (1.11–1.82), p = 0.005) could be detected.

**Table 3:** Predictive value of serum cytokine levels for extrinsic asthma, allergic rhinitis, eczema, actual medication and hospitalization frequency.

log. values	extrinsic asthma	allergic rhinits	eczema	steroid use	hospitalization
eotaxin	0.38 (0.03–4.19)	1.88 (0.81–4.36)	0.41 (0.14–1.25)	0.35 (0.12–1.02)	0.50 (0.20–1.24)
	**0.426**	**0.143**	**0.116**	**0.054**	**0.136**
	*1*	*1*	*1*	*0.648*	*1*
GM-CSF	1.08 (0.55–2.09)	1.20 (0.93–1.55)	1.20 (0.88–1.65)	0.85 (0.63–1.15)	0.86 (0.66–1.12)
	**0.827**	**0.169**	**0.254**	**0.299**	**0.254**
	*1*	*1*	*1*	*1*	*1*
IFNγ	0.55 (0.23–1.35)	1.32 (0.95–1.85)	1.20 (0.79–1.82)	0.70 (0.47–1.05)	1.01 (0.71–1.42)
	**0.194**	**0.099**	**0.401**	**0.083**	**0.974**
	*1*	*1*	*1*	*0.996*	*1*
IL-4	1.46 (0.92–2.33)	1.20 (0.99–1.45)	1.15 (0.91–1.47)	0.93 (0.75–1.15)	0.95 (0.79–1.16)
	**0.107**	**0.059**	**0.238**	**0.506**	**0.632**
	*1*	*0.708*	*1*	*1*	*1*
IL-5	1.96 (1.09–3.52)	1.42 (1.11–1.82)	1.16 (0.85–1.60)	1.10 (0.85–1.44)	0.87 (0.68–1.10)
	**0.025**	**0.005**	**0.354**	**0.473**	**0.245**
	*0.300*	*0.060*	*1*	*1*	*1*
IL-8	0.95 (0.47–1.92)	1.29 (0.95–1.74)	1.13 (0.76–1.70)	1.10 (0.79–1.55)	0.81 (0.59–1.10)
	**0.891**	**0.098**	**0.544**	**0.563**	**0.180**
	*1*	*1*	*1*	*1*	*1*
IL-10	1.66 (0.64–4.26)	0.95 (0.65–1.40)	1.18 (0.71–1.97)	1.43 (0.93–2.21)	0.82 (0.55–1.22)
	**0.295**	**0.805**	**0.522**	**0.107**	**0.319**
	*1*	*1*	*1*	*1*	*1*
IL-12(p40)	2.85 (1.19–6.84)	1.11 (0.80–1.54)	0.91 (0.60–1.40)	0.92 (0.64–1.33)	1.00 (0.72–1.40)
	**0.019**	**0.525**	**0.681**	**0.660**	**0.990**
	*0.228*	*1*	*1*	*1*	*1*
IL-13	0.87 (0.50–1.52)	1.11 (0.90–1.37)	1.12 (0.85–1.49)	0.93 (0.73–1.17)	0.80 (0.64–1.00)
	**0.622**	**0.347**	**0.421**	**0.528**	**0.050**
	*1*	*1*	*1*	*1*	*0.600*
IL-17	0.63 (0.30–1.33)	1.24 (0.93–1.66)	0.95 (0.66–1.37)	0.87 (0.63–1.21)	1.00 (0.74–1.34)
	**0.226**	**0.137**	**0.803**	**0.416**	**0.988**
	*1*	*1*	*1*	*1*	*1*
TNFα	1.82 (0.25–13.14)	1.00 (0.50–2.00)	1.15 (0.44–3.05)	1.49 (0.65–3.41)	0.66 (0.31–1.42)
	**0.554**	**0.775**	**0.346**	**0.293**	**0.293**
	*1*	*1*	*1*	*1*	*1*
IFNγ/IL-5	0.36 (0.19–0.70)	0.84 (0.67–1.05)	0.93 (0.71–1.23)	0.76 (0.59–0.98)	1.15 (0.92–1.44)
	**0.003**	**0.120**	**0.627**	**0.035**	**0.234**
	*0.036*	*1*	*1*	*0.420*	*1*

Only asthmatic children (n = 443) were included in this analysis. Depicted are odds ratios followed by p-values (in bold type), and Bonferroni adjusted p-values (in italics).

Generalized estimating equations of serum cytokine measurements to clinical parameters as well as to atopic diseases, medication, and hospitalization were carried out to adjust for intra-cluster (intra-household) correlation (Table 4 and 5). In Figure S2 (supplementary data) a regression analysis was carried out to identify relations between cytokines. Positive correlations could be found between IFNγ and IL-17 levels (r = 0.67), within the T_h_2 cytokines IL-4 and IL-5 (r = 0.55), and between IFNγ and GM-CSF (r = 0.54). The division into healthy and asthmatic individuals resulted in similar correlation patterns (data not shown). Figure S3 (supplementary data) shows a principal component analysis of cytokine concentrations.

**Table 4:** Generalized estimating equations of serum cytokines and clinical characteristics.

logarithmic values	lymphocyte counts	eosinophil counts	neutrophil counts	total IgE	IgE for D1	IgE for GX1	attack frequency	asthma onset	FEV1	BHR
eotaxin	0,022	−0,338	0,280	−0,182	−0,419	−0,216	−0,121	0,855	−0,005	0,015
	**0,078**	**0,035**	**0,875**	**0,087**	**0,000**	**0,043**	**0,403**	**0,055**	**0,736**	**0,956**
GM-CSF	−0,001	0,022	0,259	0,024	−0,015	0,030	0,015	0,225	−0,004	−0,103
	**0,757**	**0,667**	**0,596**	**0,343**	**0,696**	**0,418**	**0,757**	**0,105**	**0,516**	**0,231**
IFNγ	0,004	−0,083	−0,049	−0,010	−0,024	0,038	0,128	−0,032	0,003	−0,029
	**0,548**	**0,128**	**0,939**	**0,776**	**0,572**	**0,356**	**0,035**	**0,849**	**0,627**	**0,810**
IL-4	−0,009	−0,063	0,478	0,013	−0,017	0,025	0,044	−0,043	0,004	−0,058
	**0,031**	**0,125**	**0,258**	**0,521**	**0,567**	**0,356**	**0,280**	**0,706**	**0,409**	**0,386**
IL-5	−0,010	−0,025	0,477	0,093	0,096	0,037	0,124	−0,165	−0,002	−0,048
	**0,035**	**0,703**	**0,378**	**0,001**	**0,016**	**0,324**	**0,006**	**0,271**	**0,816**	**0,593**
IL-8	−0,024	0,090	0,441	0,033	−0,045	0,147	−0,138	0,400	0,016	0,179
	**0,000**	**0,237**	**0,630**	**0,330**	**0,371**	**0,005**	**0,017**	**0,029**	**0,211**	**0,326**
IL-10	−0,008	0,024	0,206	0,045	−0,020	0,037	0,056	−0,126	0,004	0,035
	**0,206**	**0,736**	**0,768**	**0,204**	**0,641**	**0,464**	**0,366**	**0,555**	**0,602**	**0,723**
IL-12 (p40)	−0,006	−0,025	0,478	0,028	0,066	−0,002	0,021	0,005	−0,003	0,152
	**0,328**	**0,711**	**0,436**	**0,436**	**0,130**	**0,956**	**0,732**	**0,983**	**0,690**	**0,111**
IL-13	0,002	−0,017	−0,330	0,004	−0,047	−0,010	0,016	−0,108	0,004	−0,030
	**0,589**	**0,695**	**0,522**	**0,870**	**0,116**	**0,748**	**0,694**	**0,338**	**0,607**	**0,700**
IL-17	−0,007	−0,082	0,575	−0,015	−0,023	0,047	0,089	−0,125	0,005	0,033
	**0,147**	**0,172**	**0,225**	**0,628**	**0,599**	**0,257**	**0,082**	**0,446**	**0,425**	**0,714**
TNFα	−0,010	0,151	−1,179	−0,081	−0,160	−0,073	−0,182	0,760	−0,024	0,291
	**0,423**	**0,233**	**0,401**	**0,324**	**0,091**	**0,524**	**0,076**	**0,032**	**0,159**	**0,163**

Depicted are regression coefficients followed by p-values (in bold type) calculated of the logarithmic values of the cytokines and clinical parameters of asthmatic children (n = 443).

**Table 5:** Generalized estimating equations of serum cytokine levels and extrinsic asthma, allergic rhinitis, eczema, actual medication and hospitalization frequency.

log. values	extrinsic asthma	allergic rhinits	eczema	steroid use	hospitalization
eotaxin	0,98 (0,94–1,02)	0,98 (0,86–1,11)	0,93 (0,81–1,07)	0,90 (0,79–1,03)	0,93 (0,80–1,08)
	**0,352**	**0,694**	**0,323**	**0,134**	**0,358**
GM-CSF	1,01(0,99–1,04)	1,02(0,99–1,06)	1,04(1,00–1,08)	0,99(0,94–1,03)	0,97(0,93–1,02)
	**0,372**	**0,230**	**0,082**	**0,557**	**0,279**
IFNγ	0,99(0,96–1,03)	1,05(1,00–1,10)	1,05(0,99–1,11)	0,95(0,90–1,01)	0,98(0,92–1,05)
	**0,724**	**0,040**	**0,113**	**0,112**	**0,582**
IL-4	1,01(1,00–1,03)	1,04(1,01–1,07)	1,01(0,97–1,05)	1,00(0,97–1,04)	0,99(0,96–1,02)
	**0,116**	**0,016**	**0,598**	**0,928**	**0,343**
IL-5	1,02(1,00–1,04)	1,07(1,03–1,11)	1,01(0,96–1,07)	1,01(0,97–1,06)	0,97(0,92–1,01)
	**0,022**	**0,001**	**0,681**	**0,486**	**0,145**
IL-8	0,99(0,97–1,02)	1,03(0,98–1,08)	1,04(0,97–1,12)	1,01(0,95–1,06)	0,97(0,91–1,02)
	**0,673**	**0,280**	**0,263**	**0,832**	**0,250**
IL-10	1,02(0,99–1,05)	1,05(0,99–1,10)	1,04(0,97–1,12)	1,06(1,00–1,13)	0,98(0,92–1,04)
	**0,221**	**0,085**	**0,233**	**0,065**	**0,444**
IL-12(p40)	1,03(1,00–1,05)	1,01(0,96–1,07)	1,00(0,93–1,06)	0,99(0,94–1,05)	1,00(0,94–1,06)
	**0,076**	**0,624**	**0,906**	**0,819**	**0,901**
IL-13	1,01(0,99–1,02)	1,03(0,99–1,06)	1,01(0,97–1,05)	1,00(0,96–1,03)	0,97(0,93–1,01)
	**0,598**	**0,099**	**0,648**	**0,898**	**0,099**
IL-17	1,00(0,97–1,02)	1,05(1,00–1,10)	1,04(0,99–1,10)	0,98(0,92–1,03)	1,00(0,95–1,05)
	**0,815**	**0,029**	**0,118**	**0,379**	**0,947**
TNFα	0,99(0,94–1,03)	0,92(0,82–1,03)	1,08(0,92–1,27)	1,05(0,94–1,16)	0,93(0,84–1,03)
	**0,545**	**0,144**	**0,365**	**0,403**	**0,156**

Depicted are odds ratios followed by p-value (in bold type). Only asthmatic children (n = 443) were included in this analysis.

## Discussion

In adults and children affected with asthma, several pathophysiological effects have been associated with imbalanced T cell activation and the presence or absence of distinct immune mediators. Thus cytokines and chemokines evoke increasing interest for diagnosis and therapy. The present study aimed at understanding the pattern of circulating cytokines in asthmatic adults and children. To assess the potential value of serum cytokines as biomarker for asthma, or certain asthma phenotypes, or prediction of exacerbation of disease, we compared serum levels of eleven distinct asthma associated mediators of 218 families where at least two children have asthma. A number of authors have studied serum cytokines in asthmatic patients [15], [19], [20], [21], [22], [23]. However, no study covered such a large set of individuals with inclusion of a large number of cytokines and chemokines.

The results presented here concern asthmatic and healthy siblings and their parents and provide reference values for serum cytokine levels of asthma families and the respective age group. Like in measurements of a similar design, the results demonstrate a high variability of serum cytokine amounts between individuals [24] and at the same time, reveal distinct characteristics in the distributions of the single cytokines and chemokines analyzed (Figure S1). No differences were observed between healthy and diseased individuals in the cytokines analyzed, except IL-8 in adults (table 1). In this analysis asthma was referred to as one disease and was not divided into distinct subgroups. Our data differ from those of the exploratory study of ten Hacken et al. who found that the median levels of serum IL-4, IL-5, and IFNγ were significantly higher in asthmatic patients than in healthy controls [25]. Instead, our results support the hypothesis of asthma not being associated with changes of serum cytokine amounts, when regarded as single disease entity. A most likely explanation could be that asthma is a complex and heterogeneous disease consisting of many phenotypes [1]. On the contrary, it might be that systemic patterns of T_h_1-, T_h_2-, T_h_17-, and Treg -cytokines in serum do not reflect the same immune imbalances proposed for the diseased organs of immune mediated disorders. In this respect, it has been suggested, that findings concerning inflammatory mediators of asthmatic patients differ in serum and bronchial lavage fluid [19]. Additionally, frequencies of cytokine producing T cells in blood do not reflect those in sputum [26]. It should be mentioned that the study design of the German Asthma Family Study may not be advantageous for comparing healthy and diseased children, due to a small proportion of healthy children (n = 56), resulting in a loss of power to discriminate between health and disease. However, since the healthy children are sharing genes and environment with the asthmatic children, the discordance for disease may be of particular interest, even with low nominal power.

Serum cytokine patterns showed marked differences between parental and pediatric asthma. In asthmatic children cytokine levels demonstrated significant elevation in IL-4, IL-5, and IL-12(p40), and a decrease in eotaxin, IL-10, and TNFα, compared to asthmatic adults (table 1). This points at age being important factors in systemic immune regulation of asthma. The impact of age was also recently shown for mitogen-stimulated cytokine production in a general population [27]. Further, our data and literature data point at the importance of evaluating circulating cytokine levels in specific asthma phenotypes. Wong et al. found higher plasma concentrations of IL-18, IL-12, IL-10, and IL-13 in allergic asthmatic patients than in healthy controls [28]. Silvestri et al. stated that, IL-8 and TNFα, but not IL-16 and IL-13, serum levels were higher in severe asthmatic compared to mild-moderate asthmatics or controls [15]. Our study includes mainly individuals from university hospitals and families with two affected children. To control for correlation across family members generalized estimating equations of serum cytokine measurements to clinical parameters as well as to atopic diseases, medication, and hospitalization were carried (Table 4 and 5). Nevertheless, the study design certainly leads to a biased sample towards children with a strong familiar/genetic component and towards children with more severe disease. But, more extreme phenotypes may be more informative for detecting any biochemical or genetic abnormalities.

The most common classification of asthma is the division into extrinsic (atopic, allergic) and intrinsic (non-allergic) phenotype. The finding that systemic levels of IL-12 (p40), IL-5, and IFNγ/IL-5 reflect the regulatory differences between the allergic and non-allergic pediatric asthma subgroups (table 3), led us to address this issue more closely. The expression of IL-12, a heteromeric protein consisting of the two subunits p35 and p40, and the monomeric and homodimeric form of p40 respectively, has proven to be associated with asthma [5]. Furthermore, anti-IL-12-p35 or anti-IL-12-p40 monoclonal antibodies were shown to abolish asthma symptoms during the challenge phase in a mouse model of experimental asthma [29]. In contrast to IL-12, a substantial body of data in mice and humans support the importance of IL-5 in asthma [3], [30]. Frequent asthma attacks were significantly associated with higher circulating IL-5 levels (Figure 2). To determine, if any of the analyzed serum cytokine levels could be used as a potential disease indicator, the diagnostic value of IL-12 (p40) and IL-5 were directly tested in a receiver operating characteristic (Figure 2A, B). IL-12 and IL-5 demonstrate only moderate marker characteristics to distinguish between the extrinsic and intrinsic asthma phenotype. The clinical use of these cytokines is still hampered by the fact that there would be too high false negatives and positives.

In the present study serum cytokine levels and its correlations to clinical symptoms, such as blood cell count, total and specific IgE, asthma onset, and pulmonary function was evaluated to assess practical applicability (table 2). Overall correlating circulating cytokine concentrations to established clinical parameters of asthma did not reveal any clear pattern. Our results may indicate that serum cytokine levels and functional tests reflect distinct aspects of the pathophysiology of asthma, and clinical symptoms occur independently of systemic cytokine levels. However, this contrasts with results of Nakamura et al. who found that plasma eotaxin levels were inversely related to percent predicted FEV_1_ and directly related to asthma diagnosis in a large population [22]. Unlike results of some exploratory studies [21], [25], [31], [32], in our study asthmatic children did not demonstrate an association of serum IFNγ to clinical manifestations of asthma, possibly due to distinct study designs and inclusion criteria for the selection of cases, again outlining the crucial role of subdividing asthma into defined phenotypes.

IL-8 is a pro-neutrophilic chemokine that is secreted by various cell types. For more than 10 years, IL-8 has been suggested to play an important role in asthma, and especially in severity of disease [11], [13], [15]. In our study, analysing IL-8 serum levels in regard to clinical parameters of asthma, demonstrated several significant results. It correlated negatively with lymphocyte counts, asthma attack frequency and positively with specific IgE for GX1 (table 2) and, additionally, IL-8 serum concentrations differed between healthy and asthmatic adults (table 1). This points towards an important systemic role for IL-8. However, our analysis was hampered by the observation that IL-8 levels of the first funding period, which took place from 1995 to 1998, were markedly lower than IL-8 concentrations of the second part of the study, which took place from 2000 to 2001. This contrasts with the homogenous measurements for the other analyzed cytokines (Figure S1). Due to the fact that all cytokines were quantified in one assay and originate of the same serum sample, which was thawed only once, we can largely exclude laboratory procedures to be responsible for this phenomenon. The only difference between samples of the first study and the second study half is the time of storage at −80°C (14-11 years versus 9-8 years). IL-8 may be the most unstable of the analyzed cytokines, which is supported by the conclusion of Friebe et al., who recently proposed that there are major differences in the stability of cytokines in blood samples [33].

For a better understanding of the regulation of cytokine networks in asthmatic patients, we considered further the possibility of a direct or inverse relationship between T_h_2, T_h_1, Treg and T_h_17 cytokines. Pair wise assessment of correlations among the analyzed cytokines, revealed a strong positive correlation between IFNγ and IL-17 within healthy and asthmatic individuals (Figure S2). This is in accordance with previous data where elevation of the dominant T_h_1 cytokine IFNγ and the strong pro-inflammatory cytokine IL-17, have been reported in sputum of asthmatic patients [26], [34]. It is obvious in our analysis that the cytokines are correlated in healthy controls and asthmatic patients. Based on the knowledge that IFNγ, has generally been considered having a negative role on T_h_17 cell differentiation and expansion [35], this direct relation could be due to an individually balanced cytokine pattern. This is favored also by a direct relation for the T_h_2 cytokines IL-4 and IL-5, and IFNγ and GM-CSF. The hypothesis of a relative balance appearing more important than actual concentrations was suggested also recently by Halonen et al. for cytokine production in mothers, fathers, and infants of a large birth cohort [27].

Our data do not provide information about the origin of the analyzed cytokines. It is known that during allergic reactions, no activated lymphocytes are found in the circulation, although there is an increase in local lymphocyte activity [36]. Such factors would favor the importance of the local environment, such as lung tissue, to search for a marker of practical applicability, but less invasive measures, such as serum, represent the great advantage of being much easier to obtain, especially in children. Considering the inconclusive overall pattern in obtaining biomarker characteristics for any of the analyzed cytokines, our data do not support the idea of a routine evaluation of serum cytokines in asthmatic patients and point at the importance of precisely defining asthma subtypes. Little is known about the dynamics of circulating cytokines, including degradation, regulation and half life, which was not addressed in this study and remain to be elucidated in further studies. This knowledge would pave the way for a conclusive interpretation and validation of systemic immune mediators and the discovery of a serological marker for asthma or asthma phenotypes or severity respectively.

In conclusion, the present study demonstrates main characteristics of serum cytokine patterns in healthy and asthmatic children and adults, and provides for the first time reference values for these groups in a large population. Serum patterns were not different between healthy and asthmatic individuals, with regard to absolute amounts. By looking at distinct phenotypes of asthma, patterns of circulating immune mediators demonstrated major differences. Serum levels of six out of eleven mediators analyzed differed in parental and pediatric asthma. IL-12 (p40), IL-5, and IFNγ/IL-5 showed differences between the extrinsic and intrinsic asthma phenotype. High IL-5 serum levels are associated with frequent asthma attacks in children. Furthermore, cytokine patterns seem to be individually balanced both in healthy and diseased adults and children. The precise mechanism of how systemic cytokines and their coactive interactions might be involved in driving asthma processes, however, remains elusive. A further expansion of the cytokine panel tested, possibly coupled even with direct local measurements and clearly defined asthma phenotypes is warranted.

## Supporting Information

Table S1 Assay Sensitivities of MILLIPLEX MAP for the 11 analyzed Cytokines and Chemokines.
(0.05 MB DOC)

Figure S1 Histogram of serum cytokine levels of all individuals. Each of the cytokines has its own scale and concentrations are in the range of pg/ml. The 944 study participants are ordered chronologically by the time of examination (on the x axis). There is a high variability of serum cytokine amounts between individuals. One individual showed extremely high levels at nearly all cytokines. Single cytokines demonstrate distinct characteristics in their distributions.
(0.38 MB TIF)

Figure S2 Correlation matrix of the analyzed cytokine concentrations. Pair wise assessment of correlations, revealed a strong positive relation for IL-17 and IFNγ levels (rs = 0.67), GM-CSF and IFNγ levels (rs = 0.54), as well as IL-4 and IL-5 (rs = 0.55).
(2.55 MB TIF)

Figure S3 Principal component analysis of serum cytokine concentrations.
(0.13 MB TIF)
