# Supplementary material for: Cytokine Profiles in Asthma Families Depend on Age and Phenotype
Source: PLoS One. 2010 Dec 13;5(12):e14299. doi: 10.1371/journal.pone.0014299 (PMC3001464; doi:10.1371/journal.pone.0014299)
Supplement: Table S1 — Assay Sensitivities of MILLIPLEX MAP for the 11 analyzed Cytokines and Chemokines. (0.05 MB DOC) [file pone.0014299.s001.doc]

| Cytokine | Mean MinDC [pg/ml] | Number of values below lower limit | Number of values above upper limit |
| --- | --- | --- | --- |
| eotaxin | 1.2 | 2 | — |
| GM-CSF | 9.5 | 100 | 14 |
| IFN- γ | 0.1 | 23 | — |
| IL-4 | 0.6 | 383 | — |
| IL-5 | 0.1 | 389 | — |
| IL-8 | 0.2 | — | 1 |
| IL-10 | 0.3 | 76 | — |
| IL-12 (p40) | 10.5 | 325 | 1 |
| IL-13 | 0.4 | 436 | — |
| IL-17 | 0.2 | 43 | — |
| TNF- α | 0.1 | — | — |
