# Supplementary figures and images for: Cytokine Profiles in Asthma Families Depend on Age and Phenotype
Source: PLoS One. 2010 Dec 13;5(12):e14299. doi: 10.1371/journal.pone.0014299 (PMC3001464; doi:10.1371/journal.pone.0014299)

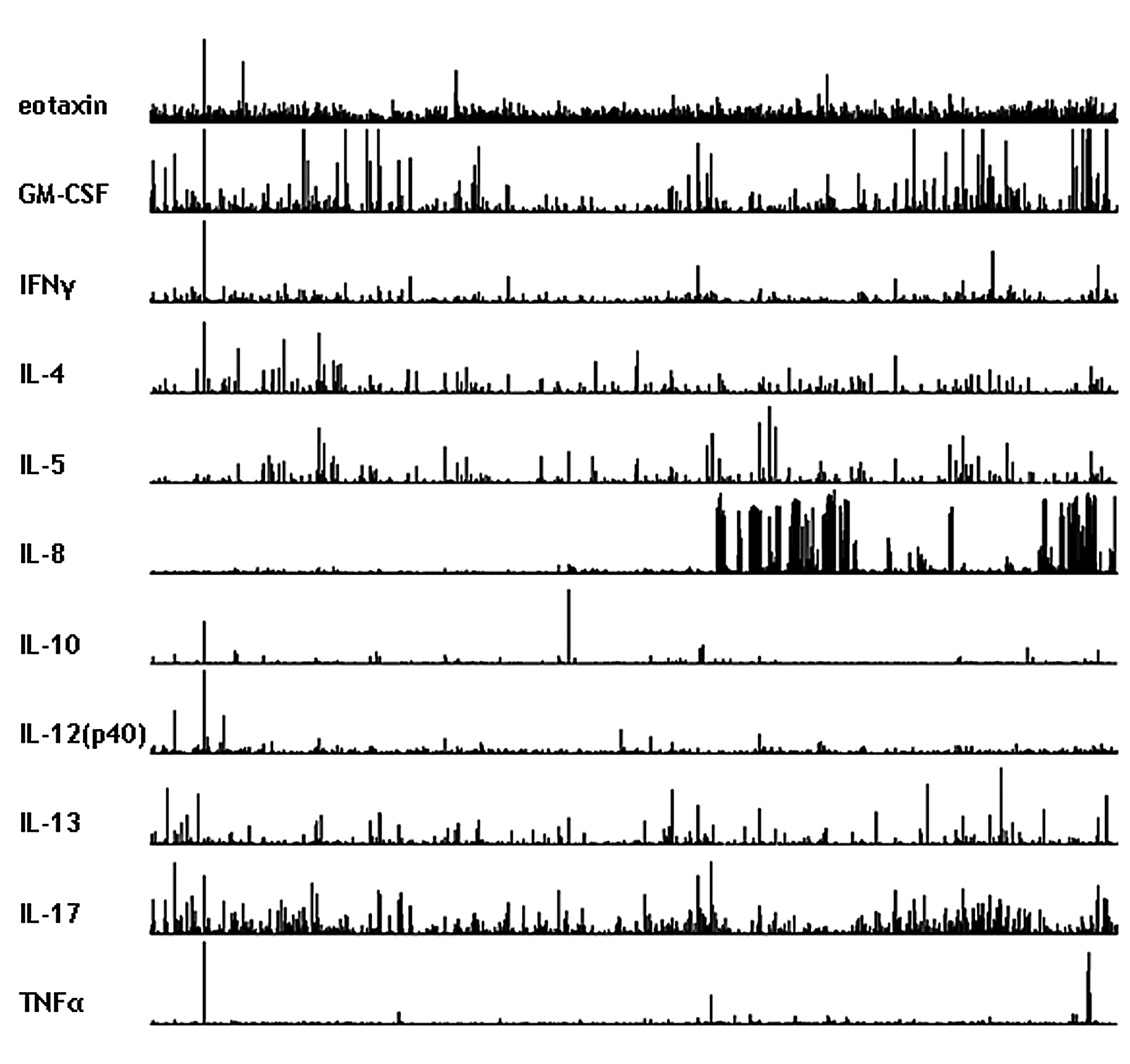

Supplement: Figure S1 — Histogram of serum cytokine levels of all individuals. Each of the cytokines has its own scale and concentrations are in the range of pg/ml. The 944 study participants are ordered chronologically by the time of examination (on the x axis). There is a high variability of serum cytokine amounts between individuals. One individual showed extremely high levels at nearly all cytokines. Single cytokines demonstrate distinct characteristics in their distributions. (0.38 MB TIF) [file pone.0014299.s002.tif]

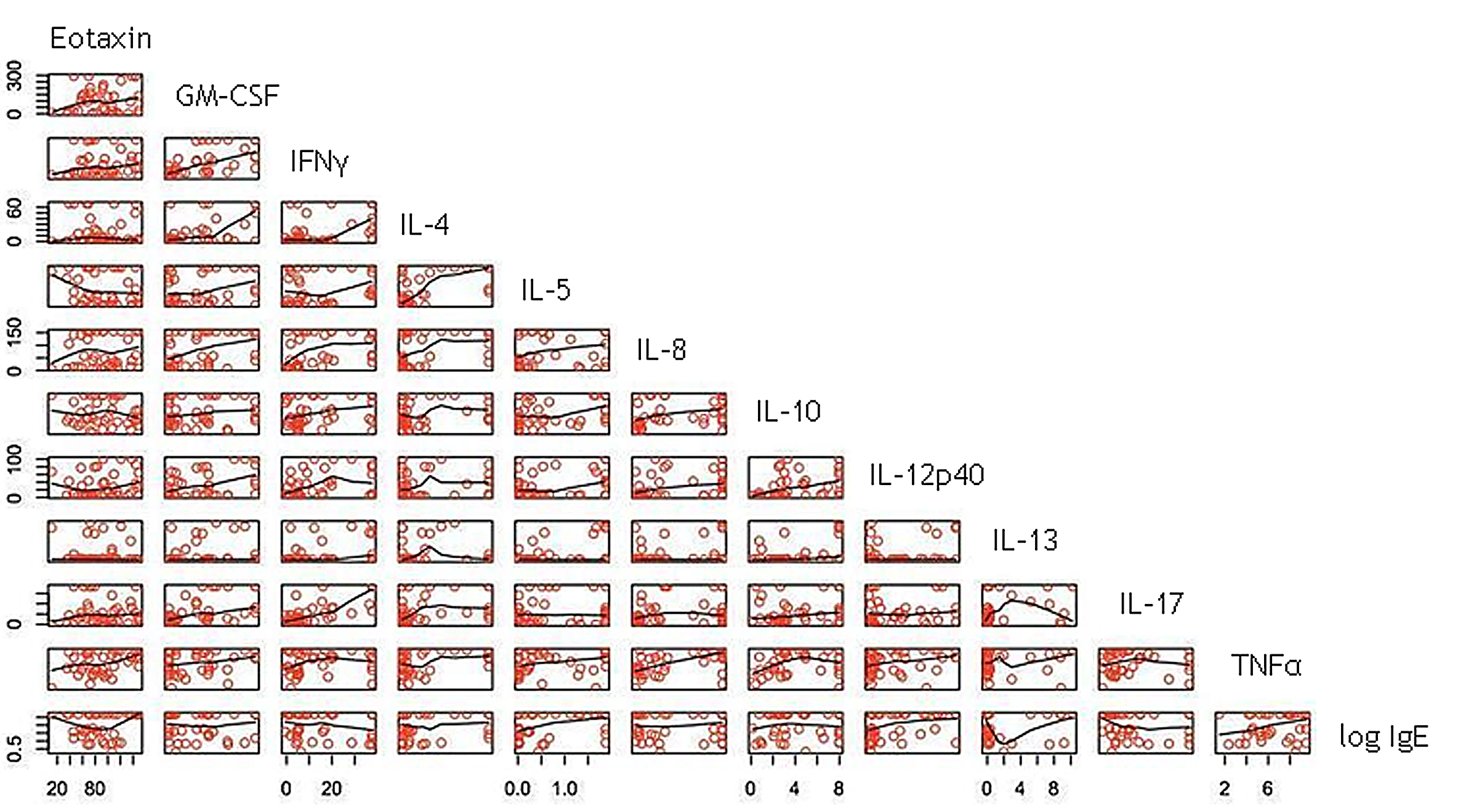

Supplement: Figure S2 — Correlation matrix of the analyzed cytokine concentrations. Pair wise assessment of correlations, revealed a strong positive relation for IL-17 and IFNγ levels (rs = 0.67), GM-CSF and IFNγ levels (rs = 0.54), as well as IL-4 and IL-5 (rs = 0.55). (2.55 MB TIF) [file pone.0014299.s003.tif]

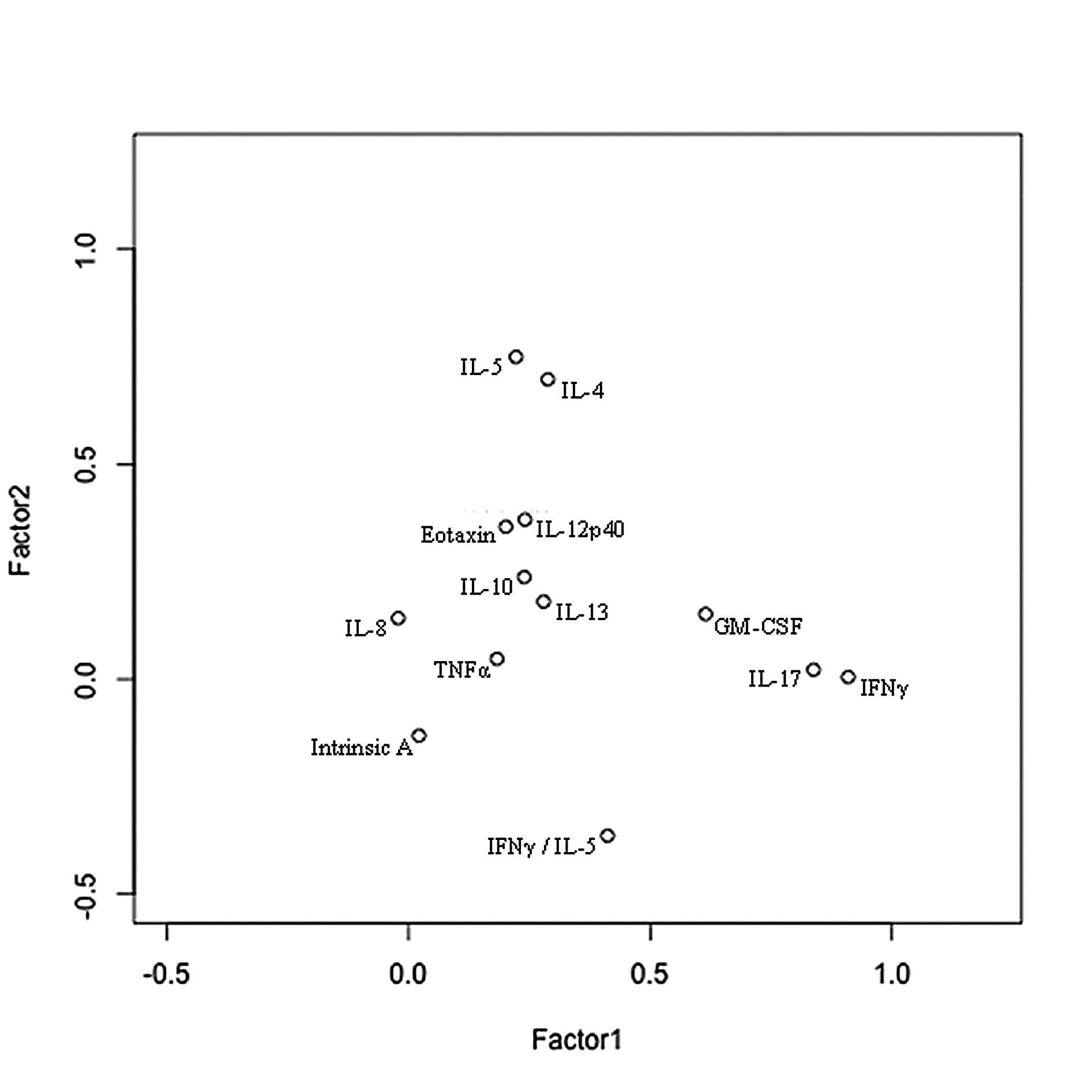

Supplement: Figure S3 — Principal component analysis of serum cytokine concentrations. (0.13 MB TIF) [file pone.0014299.s004.tif]
